# A Validated Stability-Indicating HPLC Method for Simultaneous Determination of Amoxicillin and Enrofloxacin Combination in an Injectable Suspension

**DOI:** 10.3390/scipharm85010006

**Published:** 2017-02-15

**Authors:** Nidal Batrawi, Shorouq Wahdan, Fuad Al-Rimawi

**Affiliations:** 1 The Advanced Veterinary Company, Ramallah,99765, Palestine; nidbat@yahoo.com (N.B.); sh_wahdan@yahoo.com (S.W.); 2 Department of Chemistry and Chemical Technology, Faculty of Science and Technology, Al-Quds University, Jerusalem 20002, Palestine

**Keywords:** amoxicillin, enrofloxacin, injectable suspension, HPLC, method development, validation

## Abstract

The combination of amoxicillin and enrofloxacin is a well-known mixture of veterinary drugs; it is used for the treatment of Gram-positive and Gram-negative bacteria. In the scientific literature, there is no high-performance liquid chromatography (HPLC)-UV method for the simultaneous determination of this combination. The objective of this work is to develop and validate an HPLC method for the determination of this combination. In this regard, a new, simple and efficient reversed-phase HPLC method for simultaneous qualitative and quantitative determination of amoxicillin and enrofloxacin, in an injectable preparation with a mixture of inactive excipients, has been developed and validated. The HPLC separation method was performed using a reversed-phase (RP)-C18e (250 mm × 4.0 mm, 5 μm) column at room temperature, with a gradient mobile phase of acetonitrile and phosphate buffer containing methanol at pH 5.0, a flow rate of 0.8 mL/min and ultraviolet detection at 267 nm. This method was validated in accordance with the Food and Drug Administration (FDA) and the International Conference on Harmonisation (ICH) guidelines and showed excellent linearity, accuracy, precision, specificity, robustness, ruggedness, and system suitability results within the acceptance criteria. A stability-indicating study was also carried out and indicated that this method can also be used for purity and degradation evaluation of these formulations.

## 1. Introduction

The combination of the two antibacterial drugs amoxicillin and enrofloxacin is a well-known mixture of veterinary drugs. In this drug, a synergistic effect has been demonstrated in vitro between quinolones and β-lactams. This drug is indicated for the treatment of Gram-positive and Gram-negative bacterial infections in the digestive tract, and in respiratory, intestinal, urinary, and skin infections in cattle and dogs [1,2,3].

**Figure 1:**
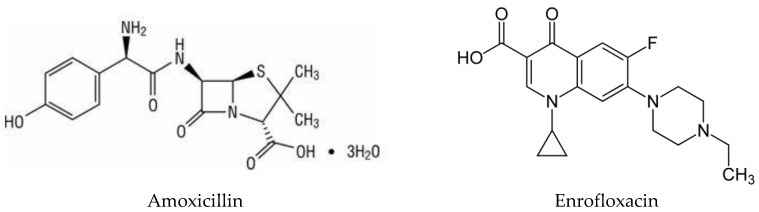
Chemical structure of amoxicillin and enrofloxacin.

Amoxicillin (α-amino-hydroxy benzyl penicillin) is a broad-spectrum penicillin categorized under the β-lactam class of antibiotics. It is a semi-synthetic antibiotic derived from a precursor molecule called 6-aminopenicillanic acid [4]. Amoxicillin is bactericidal in action and interferes with cell wall synthesis in bacteria by inhibiting cross-linking of peptidoglycan molecules, which is a cell wall component in Gram-positive (major) and Gram-negative bacteria. It is effective against *Staphylococcus* spp., *Streptococcus pneumonia*, *Streptococcus* spp., *Enterococcus faecalis*, *Escherichia coli*, *Helicobacter pylori*, *Neisseria gonorrhoeae*, *Haemophilus influenza*, *Proteus mirabilis* [4]. Enrofloxacin is a quinolone carboxylic acid derivative with antimicrobial action. It is effective against a broad spectrum of Gram-negative bacteria and is indicated for infections of the respiratory, gastrointestinal and urinary tracts in cattle, pigs and poultry. Enrofloxacin is bactericidal through the inhibition of DNA-gyrase [5]. The structure of amoxicillin trihydrate and enrofloxacin is shown in Figure 1.

In the literature, there are many analytical methods for the individual determination of amoxicillin trihydrate or enrofloxacin [4,5,6,7,8,9,10,11,12,13,14], but not for both of them simultaneously, by high-performance liquid chromatography (HPLC)-UV. The objective of this study was therefore to develop and validate a simple reverse-phase (RP)-HPLC method using a UV-PDA (photodiode array) detector to simultaneously quantify amoxicillin and enrofloxacin. This method can be used for the assay of both active ingredients in a single run. In addition, the method is stability-indicating, which provides a high degree of analytical confidence, and it can specifically detect any degradation product that may be produced during the study of stability or during its shelf life. This method was validated in accordance with the requirements of Food and Drug Administration (FDA) and thr International Conference on Harmonisation (ICH) guidelines [15,16,17,18,19].

## 2. Materials and Methods

### 2.1. Instrumentation

Chromatographic analysis was carried out using Dionex-Ultimate 3000 HPLC system equipped with LPG-3400SD pump, WPS-3000SL autosampler, TCC-3000 column oven, and DAD-3000 UV-VIS diode array detector (Thermo Fisher Scientific, Waltham, MA, USA). Chromeleon Data system Software (Version 6.80 DU10A Build 2826 (171948) Thermo Fisher Scientific) was used for data acquisition and mathematical calculations.

### 2.2. Materials and Reagents

Amoxicillin and enrofloxacin active ingredients as reference standards were purchased from USP (Rockville, MD, USA). The injectable dosage form amoxicillin (as trihydrate) 100 mg per mL and enrofloxacin 50 mg per mL was formulated in house in R&D laboratory of the company. The methanol and acetonitrile used were of HPLC grade and obtained from Merck (Darmstat, Germany). Water for HPLC analysis was obtained by double distillation prepared by Aquatron equipment model A 4000D (Bibby Sterilin Ltd, Stone, UK). Other reagents such as potassium dihydrogen phosphate, phosphoric acid, hydrochloric acid, sodium hydroxide, and hydrogen peroxide were purchased from Merck (Darmstat, Germany), Sigma Aldrich (Billerica, MA, USA) and J. T. Baker (Phillipsburg, NJ, USA).

### 2.3. Chromatographic Conditions

Buffer and acetonitrile were used in the preparation of the diluent (75% Buffer: 25% Acetonitrile) and the mobile phase was run as gradient elution as follows: 95% buffer was run for one minute and then descended to 75% in one minute, and stayed there for six minutes, and then back to the initial conditions (95% buffer) in five minutes, so 13 min is needed for complete elution of the two drugs and degradation products. The HPLC system was equilibrated for five minutes with the initial conditions (95% buffer) before injecting next sample.

The buffer used was prepared by mixing 75 mL methanol with 425 mL of 0.02 M KH_2_PO_4_, then adjusted to pH 5.0 with 2 M H_3_PO_4_. The chromatographic conditions were run as follows: flow rate: 0.8 mL/min, wavelength: 267 nm, column temperature: 25 °C, injection volume: 20 µL.

### 2.4. Preparation of Standard Solution

A standard solution of amoxicillin (0.8 mg/mL) and enrofloxacin (0.4 mg/mL) was prepared by dissolving an accurately weighed amount of amoxicillin trihydrate (114.8 mg which is equivalent to 100 mg amoxicillin) and 50 mg of enrofloxacin in 50 mL of 0.01 M HCl, then 10 mL of the resulting solution was diluted to 25 mL by the diluent.

### 2.5. Preparation of Sample Solution

A sample solution from the suspension of amoxicillin and enrofloxacin was prepared with a concentration equivalent to that in standard solution (0.8 mg/mL of amoxicillin and 0.4 mg/mL of enrofloxacin) by transferring 2 mL of the suspension and about 200 mL of a mixture of 75% buffer and 25% acetonitrile into a 250-mL volumetric flask, the solution was sonicated with frequent shaking for five minutes, and then the volume was completed to mark by the same mixture.

### 2.6. Method Validation

The method was validated according to ICH and FDA guidelines for specificity, linearity, range, accuracy, precision, limit of detection (LOD)/limit of quantification (LOQ), ruggedness, and robustness [15,16,17,18,19].

#### 2.6.1. Specificity

Forced degradation was carried by exposing samples of the drug substance and drug product to stress conditions of hydrolysis, oxidation, photo and thermal; the time and condition are illustrated in Table 1. Stressed samples were analyzed occasionally; related peaks were checked for the retention times, peaks interference, spectra purity and separation factors.

#### 2.6.2. Linearity

To evaluate the linearity and range of the method, five different concentrations of amoxicillin (240, 320, 400, 480 and 560 µg/mL) and enrofloxacin (480, 640, 800, 960 and 1120 µg/mL) were prepared. Three injections from each concentration were analyzed under the same conditions.

#### 2.6.3. Accuracy and Precision

The accuracy and precision of the method were performed on three concentrations around the test concentration (80%, 100% and 120%) by nine determinations (three replicates of each concentration). The percentage recovery and relative standard deviation (RSD) were calculated for each of the replicate samples.

#### 2.6.4. Limit of Detection and Limit of Quantification

LOD and LOQ of amoxicillin and enrofloxacin using this method was determined by analyzing different dilute solutions of amoxicillin and enrofloxacin, and measuring signal to noise ratio. The limit of detection is the concentration that gives a signal to noise ratio of ≥3, while the limit of quantification in sample can be determined with acceptable precision and accuracy with a signal to noise ratio of ≥10.

#### 2.6.5. Ruggedness and Robustness

The robustness of the method was determined by analyzing samples of the drug product and standard solution using minor changes of the method conditions: mobile phase pH, detection wavelength and flow rate. Ruggedness of the method was investigated by studying the effect of different elapsed assay times and different lab analysts on the method performance. The applied raggedness and robustness parameters are illustrated in Table 2.

## 3. Results and Discussion

### 3.1. Method Development

**Figure 2:**
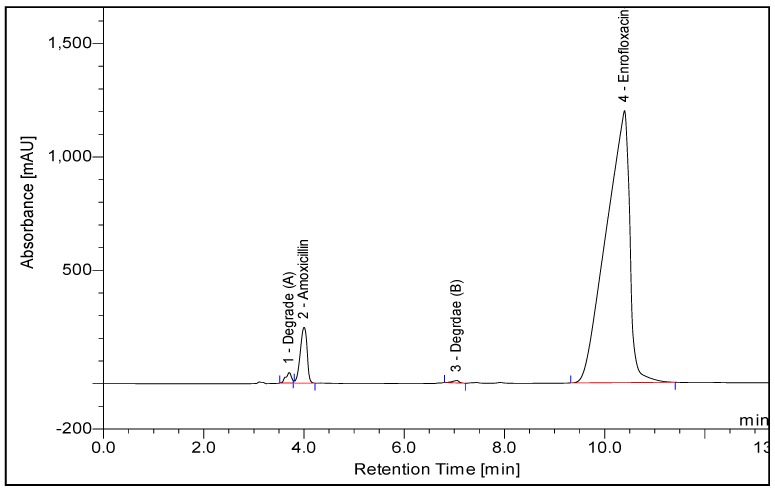
Chromatogram of well-separated peaks of the active ingredients (amoxicillin and enrofloxacin) and the degradative materials. Concentration of amoxicillin and enrofloxacin is 0.8 and 0.4 mg/mL, respectively. Degrade A and B are degradants from Amoxicillin.

Preliminary studies involved trying different stationary phases and testing several mobile phase compositions for the effective separation of amoxicillin and enrofloxacin. Method development started with testing three reversed-stationary phases (C4, C8, and C18 columns). Both analytes have retention using all these stationary phases, but for good separation of the two analytes and the degradation products as shown in Figure 2, stationary phase C18e (250 mm × 4.0 mm, 5 μm) was found to be the best one for optimum separation, as shown in Figure 2. Regarding the mobile phase, a mixture of acetonitrile and phosphate buffer containing methanol at pH 5.0 was tested both in isocratic and gradient elution. Isocratic elution was not successful for the separation of the two analytes and the degradation products even when using a high percentage of buffer. Therefore, the gradient elution was used and optimized as reported in Section 2.3. Regarding the pH of the buffer, different pH values were tested and we found that pH 5 was the best as it gave a better separation of the two analytes and degradation products. Different flow rates of 1.2, 1.0, and 0.8 mL/min were tested, and we found that 0.8 mL/min was the best one. Room temperature was good for this separation and so it was used in the whole separation. Ultraviolet detection at 267 nm was used as it was found to be the optimum wavelength for the two analytes (amoxicillin and enrofloxacin) as it gave a high signal-to-noise ratio and a high peak area for the two analytes. Using these conditions, good separation of the two analytes and the degradation products was obtained (see Figure 2), and the diluent and placebo (mixture of excipients) did not show a response in the region of the analytes.

### 3.2. Linearity and Range

The linearity of the method was observed in the concentration range of 480 µg/mL to 1120 µg/mL for amoxicillin and 240 µg/mL to 560 µg/mL for enrofloxacin, demonstrating its suitability for analysis. The goodness of fit (*R*^2^) was found to be 0.9999 and 1.0000 for amoxicillin and enrofloxacin, respectively, indicating a linear relationship between the concentration of the analytes and the area under the peak.

### 3.3. Specificity and Stability-Indicating Study

Specificity is the ability of the analytical method to measure the active ingredient response in the presence of other excipients, impurities and any potential degradants. Forced degradation was carried out to evaluate the specificity and stability-indicating properties of the method, by exposing samples of the drug substance and the drug product to conditions of hydrolysis, oxidation, photo and thermal stresses.

Stress testing of the drug product was performed to induce forced degradation and to identify the potential degradant products, the stability of the drug substance and also to validate the specificity of the analytical procedures. The stability-indicating study was performed under the various stress conditions mentioned in Section 2.6.1.

The acidic condition applied on the active drug substances for four hours induced the hydrolysis of amoxicillin, causing an assay loss of about 26% and degradative materials (A) and (B) of about 17% and 7.5%, respectively, while no degradation was observed for enrofloxacin. There was no evidence of degradation of the drug substances when exposed to alkaline- and oxidative-type stresses. There was no evidence of degradation of the drug product when exposed to thermal, oxidative and photo stress conditions.

The results showed no interference between the chromatographic peaks of amoxicillin and enrofloxacin and the excipients, impurities and degradation products under the various stress conditions (Figure 2). The spectra of all the peaks were checked using PDA, showing perfect purity.

### 3.4. Accuracy and Precision

The results of accuracy and precision testing showed that the method is accurate and precise within the acceptable limits. The percentage recovery and RSD were calculated for both active ingredients, amoxicillin and enrofloxacin, and all the results were within the limits. Acceptable accuracy was within the range of 98.0% to 102.0% recovery, and the precision of the %RSD was not more than 2.0%, as demonstrated in Table 3.

### 3.5. Ruggedness and Robustness

The ruggedness and robustness of the method were examined using the minor modifications detailed in Table 2. The data obtained indicated that minor modifications of the experimental parameters do not affect the assay and its ability to accurately and precisely detect/quantify the active ingredients.

### 3.6. Limit of Detection and Limit of Quantification

The limit of detection is the lowest amount of analyte in a sample that can be detected, but not necessarily quantitated. It may be expressed as a concentration that gives a signal-to-noise ratio of approximately 3:1. The limit of quantification is the lowest amount of analyte in a sample that can be quantitatively determined with suitable accuracy and precision, with a signal-to-noise ratio of approximately 10:1. The method showed LOQs of 6.9 and 0.24 mg/L for amoxicillin and enrofloxacin, respectively; and LODs of 2.0 and 0.074 mg/L for amoxicillin and enrofloxacin, respectively.

### 3.7. System Suitability

System suitability parameters were measured to verify the system, method, and column performance by evaluation of the column efficiency and the ability to separate peaks. Column efficiency is usually represented by the number of theoretical plates for each peak. According to FDA regulations, the number of theoretical plates for each peak must not be less than 1000 to have good separation [15]. In addition to the theoretical plates, the tailing factor is another parameter of system suitability which reflects the symmetry of the peak. The peak should be symmetrical with minimal peak broadening or fronting, which should be less than 2.0 according to FDA regulations [15]. The resolution between the adjacent peaks is another parameter of system suitability where good resolution is required to obtain good separation of the adjacent peaks. According to the FDA regulations, resolution should be not less than 1.5 [15].

The current method shows that all the values for the system suitability parameters are within the acceptable limits. The column efficiencies were 9671 and 3372 theoretical plates for amoxicillin and enrofloxacin, respectively. The tailing factors were 1.11 and 0.93 for amoxicillin and enrofloxacin, respectively. The resolution between amoxicillin and enrofloxacin was 3.8.

## 4. Conclusions

A simple, accurate, and precise stability-indicating HPLC method was developed and validated for routine qualitative and quantitative analysis of amoxicillin and enrofloxacin in an injectable formulation.

The method is stability-indicating, and therefore qualified and reliable for demonstrating and detecting any expected change or degradation in the drug product during stability studies. The method is robust and rugged enough to reproduce accurate and precise results under different method conditions.

## Figures and Tables

**Table 1:** Stress conditions applied for drug substance and drug product.

Stress Type	Conditions	Time
Acid hydrolysis	2 mg/mL in 0.1 N HCl at RT	4 h
Base hydrolysis	2 mg/mL in 0.1 N (up to 1 N), NaOH at 65 °C	7 days
Oxidative/solution	0.3% H_2_O_2_; at RT; protected from light	7 days
Thermal	70 °C	3 weeks
Photo-degradation	UV light	3 days

RT: room temperature.

**Table 2:** The applied ruggedness and robustness conditions for the method of determination of amoxicillin and enrofloxacin.

Robustness/Ruggedness Parameter	Conditions Checked
pH of the mobile phase	4.8, 5.0 & 5.2
Detection wavelength	265, 267 and 269 nm
Flow rate	0.7, 0.8 and 0.9 mL/min
Elapsed assay times	The same analyst analyzed the same trial in two different days. Same trial was analyzed at different times after preparing the sample solution.
Different lab analysts	Two lab analysts analyzed the same trial in the same day.

**Table 3:** Accuracy and precision results of amoxicillin (A) and enrofloxacin (B).

**(A)**							
**Amoxicillin**	**Sample Peak Area**			**Standard Peak Area**			**Assay (%)**
**Sample No.**	**inj # 1**	**inj # 2**	**Average**	**inj # 1**	**inj # 2**	**Average**	
80%							
1	43.30	43.30	43.30	43.30	43.30	43.30	100.00
2	42.70	42.70	42.70				98.61
3	43.10	43.10	43.10				99.54
100%							
1	53.80	53.70	53.75	53.00	53.50	53.25	100.94
2	54.10	54.10	54.10				101.60
3	53.40	53.30	53.35				100.19
120%							
1	63.90	63.90	63.90	63.60	63.00	63.30	100.95
2	63.80	63.80	63.80				100.79
3	64.00	64.10	64.05				101.18
Mean							100.42
SD							0.93
RSD							0.93
**(B)**							
**Enrofloxacin**	**Sample Peak Area**			**Standard Peak Area**			**Assay (%)**
**Sample No.**	**inj # 1**	**inj # 2**	**Average**	**inj # 1**	**inj # 2**	**Average**	
80%							
1	519.80	519.00	519.40	511.40	513.40	512.40	101.37
2	512.00	512.20	512.10				99.94
3	517.30	518.30	517.80				101.05
100%							
1	667.50	668.30	667.90	655.50	656.10	655.80	101.85
2	674.30	673.60	673.95				102.77
3	664.30	666.90	665.60				101.49
120%							
1	789.60	791.40	790.50	788.20	782.90	785.55	100.63
2	794.90	795.90	795.40				101.25
3	799.10	798.10	798.60				101.66
Mean							101.33
SD							0.79
RSD							0.78

Inj: injection; RSD: relative standard deviation.
